# First mitochondrial genome from Yponomeutidae (Lepidoptera, Yponomeutoidea) and the phylogenetic analysis for Lepidoptera

**DOI:** 10.3897/zookeys.879.35101

**Published:** 2019-10-09

**Authors:** Mingsheng Yang, Bingyi Hu, Lin Zhou, Xiaomeng Liu, Yuxia Shi, Lu Song, Yunshan Wei, Jinfeng Cao

**Affiliations:** 1 College of Life Science and Agronomy, Zhoukou Normal University, Zhoukou, Henan, 466000, China Zhoukou Normal University Zhoukou China; 2 Chifeng Agricultural and Animal Husbandry Scientific Research Institute, Chifeng, Neimenggu, 024031, China Chifeng Agricultural and Animal Husbandry Scientific Research Institute Chifeng China; 3 Cangzhou Academy of Agriculture and Forestry Sciences, Cangzhou, Hebei, 061001, China Cangzhou Academy of Agriculture and Forestry Sciences Cangzhou China

**Keywords:** Mitogenome evolution, next-generation sequencing, protein-coding genes

## Abstract

The complete mitochondrial genome (mitogenome) of *Yponomeuta montanatus* is sequenced and compared with other published yponomeutoid mitogenomes. The mitogenome is circular, 15,349 bp long, and includes the typical metazoan mitochondrial genes (13 protein-coding genes, two ribosomal RNA genes, and 22 transfer RNA genes) and an A + T-rich region. All 13 protein-coding genes use a typical start codon ATN, the one exception being *cox1*, which uses CGA across yponomeutoid mitogenomes. Comparative analyses further show that the secondary structures of tRNAs are conserved, including loss of the Dihydorouidine (DHU) arm in *trnS1* (AGN), but remarkable nucleotide variation has occurred mainly in the DHU arms and pseudouridine (TψC) loops. A + T-rich regions exhibit substantial length variation among yponomeutoid mitogenomes, and conserved sequence blocks are recognized but some of them are not present in all species. Multiple phylogenetic analyses confirm the position of *Y. montanatus* in Yponomeutoidea. However, the superfamily-level relationships in the Macroheterocera clade in Lepidoptera recovered herein show considerable difference with that recovered in previous mitogenomic studies, raising the necessity of extensive phylogenetic investigation when more mitogenomes become available for this clade.

## Introduction

The mitochondrial genome (mitogenome) is a circular and double-stranded molecule that usually encodes 37 genes (13 protein-coding genes (PCGs), two ribosomal RNA genes (rRNAs), 22 transfer RNA genes (tRNAs)), and an A + T-rich region (Boore 1999). Characterized by cellular abundance, absence of introns, rapid evolution, and a lack of extensive recombination, mitogenome sequences can be easily amplified and has been extensively employed in evolutionary studies in past decades (Cameron 2014; Curole et al. 2014). Additionally, the mitogenome often exhibits some characters such as gene rearrangement that have been widely used to infer genome evolution and phylogeny for multiple groups. For instance, comparative analyses of lepidopteran mitogenomes showed the gene order *trnM*-*trnI*-*trnQ* is present in more derived ditrysian lineage and its close relatives Tischerioidea and Palaephatoidea, in contrast to the ancestral *trnI*-*trnQ*-*trnM* in other lepidopterans such as *Thitarodes renzhiensis* (Yang, 1991) and *T. yunnanensis* (Yang, 1992) (Cao et al. 2012; Timmermans et al. 2014).

Lepidoptera is the second largest insect order after Coleoptera, with more than 157,000 extant species in 43 superfamilies (van Nieukerken et al. 2011; Mitter et al. 2017). To date, mitogenomes of more than 400 lepidopteran species or subspecies have been sequenced (https://www.ncbi.nlm.nih.gov; last visited on March 2019). Relative to other species-rich orders, however, the current number of sequenced mitogenomes is still limited. Moreover, deep-level lepidopteran phylogeny is still poorly understood despite previous investigations based on various data including mitogenome sequences (Mitter et al. 2017). The superfamily Yponomeutoidea, with approximately 1,800 described species, represents one of the earliest diverging lineages of ditrysian Lepidoptera and includes many notable pest species (van Nieukerken et al. 2011; Sohn et al. 2013). In Yponomeutoidea, 11 families were recognized based on a multiple-gene dataset (Sohn et al. 2013), but phylogenetic relationships among yponomeutoid families still need further investigation (Mitter et al. 2017). To date, mitogenomes of only three yponomeutoid species representing three families have been published. According to the classification system proposed by Sohn et al. (2013), they are *Prays oleae* (Bernard, 1788) (Praydidae) (van Asch et al. 2016), *Plutella xylostella* (Linnaeus, 1758) (Plutellidae) (Wei et al. 2013; Dai et al. 2016), and *Leucoptera malifoliella* (Costa, 1836) (Lyonetiidae) (Wu et al. 2012). Thus, the number of reported yponomeutoid mitogenomes is quite limited. Moreover, a comparative analysis among the published mitogenomes has never been conducted.

Mitogenomic data of major Yponomeutoidea lineages would play an important role for better understanding the evolution of the superfamily or even Lepidoptera as a whole. In the present study, we sequenced the complete mitogenome of *Yponomeuta montanatus* Moriuti, 1977, the first mitogenome from the family Yponomeutidae. Moreover, detailed comparative analyses were conducted based on this and all other published yponomeutoid mitogenomes. In addition, extensive phylogenetic analyses using three different datasets and three different tree-constructed methods were performed to test phylogenetic implications of the *Y. montanatus* mitogenome in Lepidoptera phylogeny. This study contributes to further understanding the mitogenome evolution and phylogeny of the Yponomeutoidea and Lepidoptera.

## Materials and methods

### Sample collection, identification and DNA extraction

Adult *Y. montanatus* specimens were sampled by light trap at Mountain Jigongshan, Henan, China in May 2018. Fresh specimens were stored in 95–100% ethanol in the field and then maintained at –80 °C until used for DNA extraction. Dry specimens were identified based on the morphological description and illustrations provided by Byun and Bae (2013). In addition, molecular identification was performed by blasting the standard *cox1* barcode sequence in GenBank. Thorax muscle tissues were used to extract genomic DNA with the DNeasy tissue kit (Qiagen, Hilden, Germany) according to the manufacturer’s instructions. Voucher specimens are deposited in the Biology Laboratory of Zhoukou Normal University, China.

### Mitogenome sequencing and assembly

Next-generation sequencing methods were used to obtain the complete mitogenome sequence of *Y. montanatus*. Briefly, total genomic DNA was firstly quantified and fragmented to an average size of 400 bases using Covaris M220 system with the Whole Genome Shotgun method (Covaris, Woburn, MA, USA). Then, a library was constructed using the TruSeq DNA PCR-Free Sample Preparation Kit (Illumina, USA). Lastly, Illumina HiSeq 2500 was used for sequencing with the strategy of 251 paired-ends.

A total of 3,707,876 raw paired reads were retrieved for *Y. montanatus*. FastQC (http://www.bioinformatics.babraham.ac.uk/projects/fastqc) was used for quality control (avg. Q20 > 95.1%, avg. Q30 > 88.65%). After processing with AdapterRemoval v. 2 (Mikkel et al., 2016) and SOAPdenovo v. 2.01 (Luo et al. 2012), the raw paired reads were filtrated into a total of 2,582,644 high-quality reads. Then, the A5-miseq v20150522 (Coil et al. 2015) and SPAdes v. 3.9.0 (Bankevich et al. 2012) were used in de novo assembly, generating contig and scaffold sequences. Lastly, the mitochondrial sequences were identified using blastn method, and the mummer v. 3.1 (Kurtz et al. 2004) was used to establish the position relationships among the contig sequences and to fill in the possible gaps.

### Mitogenome annotation and comparative analysis

The MITOS webserver was employed to annotate the complete mitogenome sequence with the invertebrate genetic code (Bernt et al. 2013). The tRNAScan-SE server v. 1.21 (Lowe and Eddy 1997) was used to re-identify the 22 tRNAs as well as to reconfirm their secondary structures. Gene boundaries were re-identified by aligning the new mitogenome with previously reported yponomeutoid mitogenomes using MEGA v. 6.06 (Tamura et al. 2013). To ensure the correct reading frame, nucleotide sequences of the 13 PCGs were translated with both the programs Primer Premier v. 5.00 (Premier Biosoft International, Palo Alto, CA) and MEGA v. 6.06 (Tamura et al. 2013) with invertebrate mitochondrial genetic code. Tandem repeat elements in the A + T-rich region were identified using the Tandem Repeats Finder program (http://tandem.bu.edu/trf/trf.html) (Benson 1999). All other published yponomeutoid mitogenomes, along with the one sequenced in this study were compiled for comparative analysis. Base composition and the relative synonymous codon usage (RSCU) were calculated using MEGA v. 6.06 (Tamura et al. 2013). Strand asymmetry was calculated according to the formulas: AT-skew = [A – T]/[A + T] and GC-skew = [G – C]/[G + C] (Perna and Kocher 1995). The nucleotide diversity and the ratio of non-synonymous substitution (Ka) to synonymous substitution (Ks) were calculated with DNASP v. 5.0 (Librado and Rozas 2009).

### Phylogenetic analyses

To investigate phylogenetic implications of the *Y. montanatus* mitogenome in Lepidoptera phylogeny, a total of 33 mitogenomes representing 15 lepidopteran superfamilies with mitogenome available (Suppl. material 1, Table S1) were sampled for phylogenetic analyses. Two additional trichopteran mitogenomes were selected as outgroups. Sequence alignment was conducted on the TranslatorX online platform (Abascal et al. 2010) for 13 PCGs. The two rRNAs and 22 tRNAs were aligned with the Q-INS-i algorithm implemented in the MAFFT online platform (Katoh et al. 2017). MEGA v. 6.06 (Tamura et al. 2013) was used to check all alignments. Then, MEGA v. 6.06 (Tamura et al. 2013) was also used to generate three different datasets: PCG123 (all codon positions of 13 PCGs), PCG123R (PCG123 dataset plus two rRNAs and 22 tRNAs), and PCGAA (amino acid sequences translated from 13 PCGs). Nucleotide sequence substitution model was selected using PartitionFinder v. 1.1.1 (Lanfear et al. 2012), with the Baysian Information Criterion (BIC) algorithm under a greedy search. The best partition scheme and corresponding models are shown in Suppl. material 1, Tables S2 and S3.

Maximum likelihood (ML) analyses were conducted using two methods. The raxmlGUI version 1.539 interface (Silvestro and Michalak 2012) of RAxML version 7.2.6 (Stamatakis 2006) was used under the GTRGAMMAI model for PCG123 and PCG123R datasets, and the model MtArt + I + G for PCGAA dataset. Node reliability was assessed using the ML + rapid bootstrap algorithm with 100 replicates. IQ-TREE 1.6.7.1 (Nguyen et al. 2015) was used with the models determined by PartitionFinder for PCG123 and PCG123R datasets, and the model MtArt + I + G for PCGAA dataset. Node support was assessed using 1,000 ultrafast bootstrap replicates.

Bayesian inference (BI) analysis was performed using MrBayes v. 3.1.2 (Ronquist and Huelsenbeck 2003). For the PCG123 and PCG123R datasets, the model determined by PartitionFinder was used, and for PCGAA dataset with the model MtRev + I + G. Two independent Markov chain Monte Carlo (MCMC) runs were performed for 1,000,000–3,000,000 generations sampling per 100 generations. The convergence between the two runs was established by the Tracer v. 1.6 (Effective sample sizes >200) (Rambaut et al. 2014). After the first 25% of the yielded trees were discarded as burn-in, a 50% majority-rule consensus tree with the posterior probability was generated from the remaining trees.

## Results and discussion

### General characteristics of the *Y. montanatus* mitogenome

**Figure 1.**
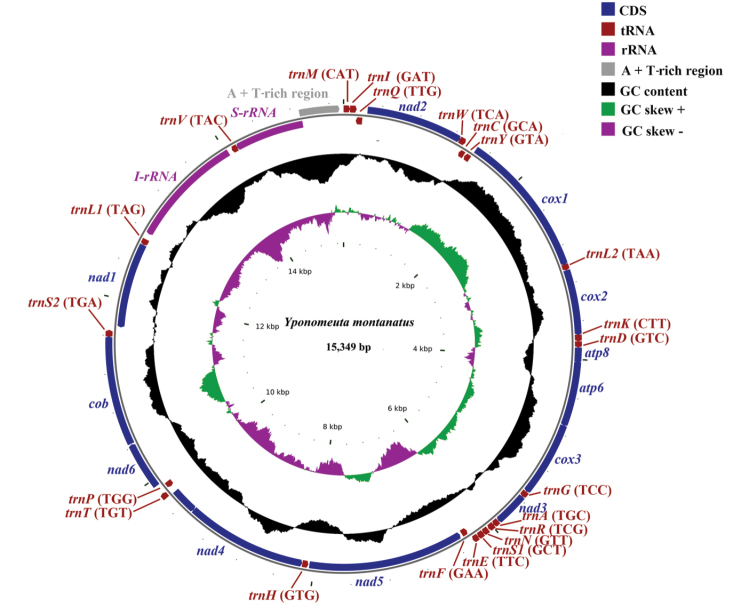
Mitochondrial genome map of the *Yponomeuta montanatus*.

The complete mitogenome of *Y. montanatus* (GenBank accession number: MK256747) is circular, double-stranded, and 15,349 bp long (Fig. 1, Table 1). This length is shortest amongst published yponomeutoid mitogenomes. The typical 37 mitochondrial genes (13 PCGs, 22 tRNAs, and two rRNAs) and an A + T-rich region are included. Among them, 23 (nine PCGs and 14 tRNAs) are encoded on majority strand (J-strand), and the remaining 14 are located on minority strand (N-strand). As in most ditrysian members of Lepidoptera, the *trnM*-*trnI*-*trnQ* can be recognized in Yponomeutoidea, in contrast to the *trnI*-*trnQ*-*trnM* in most non-ditrysian lineage such as the Hepialoidea (Cao et al. 2012), and in the ancestral arthropod mitogenome (*Drosophila yakuba*) (Clary and Wolstenholme 1985).

**Table 1.** Summary of the *Yponomeuta montanatus* mitogenome.

**Feature**	**Strand**	**Location**	**Size (bp)**	**Start codon**	**Stop codon**	**Anticodon**	**Intergenic nucleotides**
*trnM*	J	1–67	67			CAT	0
*trnI*	J	68–136	69			GAT	–3
*trnQ*	N	134–202	69			TTG	49
*nad2*	J	252–1265	1014	ATT	TAA		–2
*trnW*	J	1264–1331	68			TCA	–8
*trnC*	N	1324–1385	62			GCA	11
*trnY*	N	1397–1460	64			GTA	2
*cox1*	J	1463–2998	1536.9	CGA	TAA		–5
*trnL2* (UUR)	J	2994–3059	66			TAA	0
*cox2*	J	3060–3744	685	ATG	T		–3
*trnK*	J	3742–3812	71			CTT	–1
*trnD*	J	3812–3876	65			GTC	0
*atp8*	J	3877–4035	159	ATT	TAA		–7
*atp6*	J	4029–4706	678	ATG	TAA		–1
*cox3*	J	4706–5497	792	ATG	TAA		2
*trnG*	J	5500–5565	66			TCC	0
*nad3*	J	5566–5919	354	ATT	TAA		2
*trnA*	J	5922–5984	63			TGC	–1
*trnR*	J	5984–6050	67			TCG	8
*trnN*	J	6059–6123	65			GTT	–1
*trnS1* (AGN)	J	6123–6188	66			GCT	0
*trnE*	J	6189–6250	62			TTC	–1
*trnF*	N	6250–6315	66			GAA	22
*nad5*	N	6338–8050	1713	ATT	TAA		12
*trnH*	N	8063–8129	67			GTG	0
*nad4*	N	8130–9468	1339	ATG	T		0
*nad4I*	N	9469–9756	288	ATG	TAA		7
*trnT*	J	9764–9828	65			TGT	0
*trnP*	N	9829–9894	66			TGG	2
*nad6*	J	9897–10430	534	ATT	TAA		9
*cob*	J	10440–11591	1152	ATG	TAA		–2
*trnS2* (UCN)	J	11590–11657	68			TGA	35
*nad1*	N	11693–12631	939	ATG	TAA		1
*trnL1* (CUN)	N	12633–12699	67			TAG	0
*rrnL*	N	12700–14073	1374				0
*trnV*	N	14072–14135	64			TAC	–1
*rrnS*	N	14135–14903	769				0
A + T-rich region		14904–15349	446				

Note: the “J” indicates the majority strand and the “N” indicates the minority strand in the strand column.

As in other insect mitogenomes (Boore 1999), high A + T content is recognized across Yponomeutoidea mitogenomes, which ranges from 81% in *P. lutella* (KM023645) to 82.5% in *L. malifoliella* (Table 2). In addition to the A + T content, AT-skew and GC-skew are also routinely used to characterize base composition of mitogenomes (Perna and Kocher 1995; Wei et al. 2010). The negligible AT-skew (0.0037) and moderate GC-skew (–0.164) (Suppl. material 1, Table S4) in *Y. montanatus* mitogenome are similar to other Lepidoptera and most insect species (Cameron and Whiting 2008).

**Table 2.** Base composition of the five sequenced yponomeutoid mitogenomes.

**Taxon**	**Size (bp)**	**A + T (%)**	**PCGs**	***rrnS* RNA**	***rrnL* RNA**	**tRNAs**	**A + T-rich region**	**GenBank accession no.**
			No. of codon A + T (%)	Size (bp) A + T (%)	Size (bp) A + T (%)	Size (bp) A + T (%)	Size (bp) A + T (%)	
** Yponomeutidae **								
*Yponomeuta montanatus*	15,349	81.08	3,727 79.6	769 85.7	1,374 85.1	1,453 80.8	446 96.2	MK256747
** Praydidae **								
*Prays oleae*	16,499	81.8	3,720 79.1	773 85	1,372 85	1,486 81.3	1,483 96.3	KM874804
** Plutellidae **								
*Plutella xylostella*	16,014	81	3,731 79.4	783 86.1	1,382 85.1	1,465 81.2	888 93.1	KM023645
*Plutella xylostella*	16,179	81.4	3,729 79.4	783 86.1	1,415 84.9	1,468 81.3	1,081 n.a.	JF911819
** Lyonetiidae **								
*Leucoptera malifoliella*	15,646	82.5	3,719 80.7	770 87.1	1,351 85.5	1,488 83.7	733 95.3	JN790955

Note: n.a. indicates not available.

### Protein-coding genes

**Figure 2.**
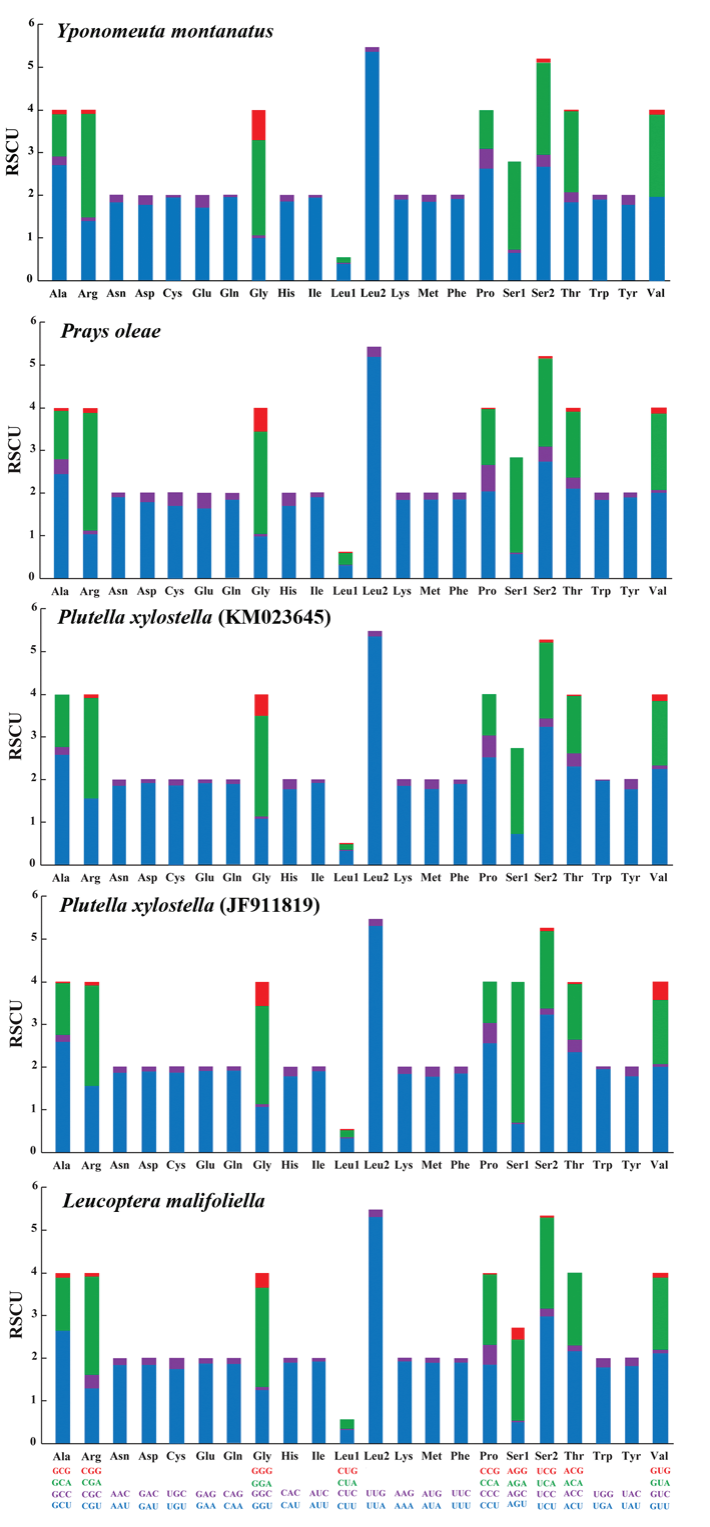
Relative synonymous codon usages (RSCU) in PCGs of *Yponomeuta montanatus* and other published yponomeutoid mitogenomes. Codon families are indicated below the X-axis.

The total length of the 13 PCGs in *Y. montanatus* mitogenome is 11,183 bp, approximately accounting for 72.9% of the whole mitogenome (Table 2). Identical to other yponomeutoid mitogenomes, nine of the 13 PCGs are encoded on J-strand, and the other four are located on N-strand. In yponomeutoid mitogenomes, the A + T content of the 13 PCGs varies from 79.1% in *P. oleae* to 80.7% in *L. malifoliella*. The codon positions show unequal A + T content (Suppl. material 1, Table S5). The third codon positions have the highest A + T content (93.4% on average), followed by first codon positions (74.9% on average) and second codon positions (70.7% on average). To characterize codon frequencies across yponomeutoid mitogenomes, relative synonymous codon usages (RSCU) were calculated and drawn for all five yponomeutoid mitogenomes. As shown in Figure 2 and Suppl. material 1, Table S6, the codon usage pattern is generally similar among them such as the most frequently used codons (i.e., UUA, AUU, UUU, AUA, and AAU). In the *Y. montanatus* mitogenome, a number of 3,727 amino acids are translated, of which 1,787 (47.9%) are encoded by the five frequently used codons above. However, the codons absent in yponomeutoid mitogenomes are different, but most of them are rich in C/G nucleotides. In general, the high A/T content in frequently used codons effectively contributes to the high A + T composition in PCGs and the whole mitogenome.

Most PCGs in yponomeutoid mitogenomes use the conventional ATN start codon (Table 1, Suppl. material 1, Table S7). The unconventional CGA was consistently found in only the *cox1* gene, a common feature for lepidopteran mitogenomes (Wu et al. 2016). TAA is employed as stop codon in most PCGs, but two other kinds of stop codons were recognized. One is the TAG for *nad4l* and *nad6* genes in *L. malifoliella*; the other is the incomplete termination codon T which is commonly used in yponomeutoid mitogenomes. Actually, the incomplete termination codon can be also commonly recognized across arthropod mitogenomes, which may be related to the post transcriptional modification during the mRNA maturation process (Ojala et al. 1981).

**Figure 3.**
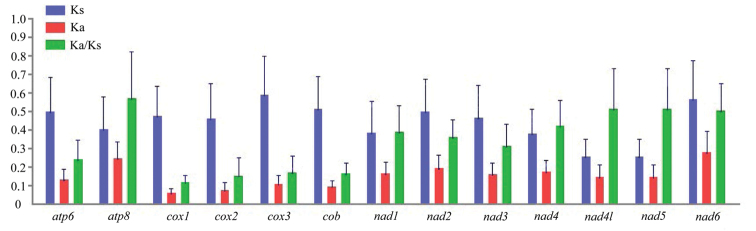
Evolutionary rate of each PCG among yponomeutoid mitogenomes. Ka, non-synonymous substitution; Ks, synonymous substitution.

To investigate evolutionary patterns of all PCGs, nucleotide diversity and the ratio of Ka to Ks were calculated for each PCG. As shown in Figure 3 and Suppl. material 1, Table S8, *nad6* and *cox1* genes show the highest and lowest nucleotide diversity respectively. The Ka/Ks values are the highest in *atp8* genes and the lowest is for *cox1* gene. Notably, the Ka/Ks values for all PCGs are lower than one, indicating that they are evolving under the purifying selection and are suitable for investigating phylogenetic relationships within Yponomeutoidea.

### Transfer and ribosomal RNA genes

**Figure 4.**
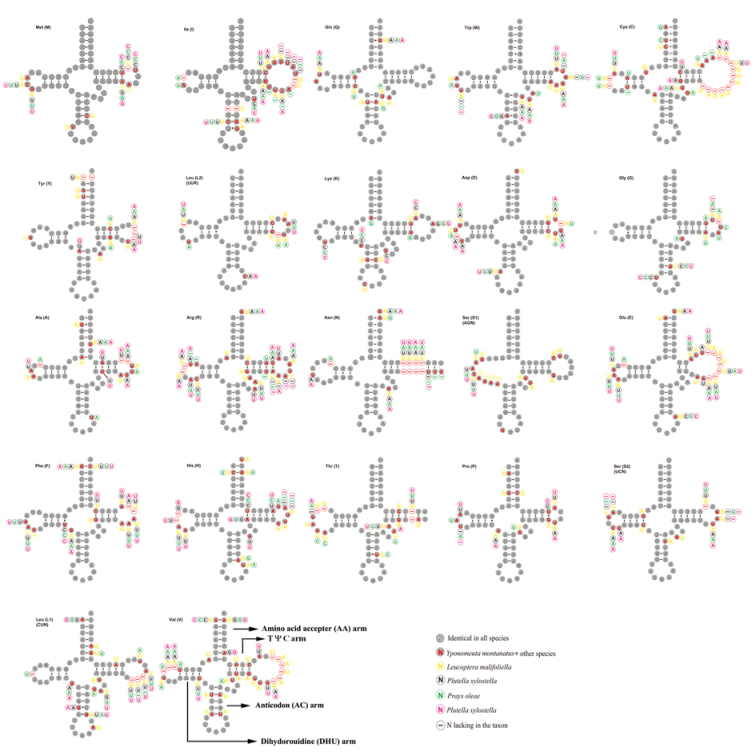
Putative secondary structures of tRNAs from *Yponomeuta montanatus* mitogenome. The tRNAs are labeled with the abbreviations of their corresponding amino acids. The tRNA arms are illustrated as for *trnV*. Dashes indicate the Watson-Crick base pairs; dots indicate the wobble GU pairs; and the other non-canonical pairs are not marked. The nucleotides marked indicate the variable sites among published yponomeutoid mitogenomes.

The *Y. montanatus* mitogenome contains 22 tRNAs with the length ranging from 62 bp (*trnC*, *trnE*) to 71 bp (*trnK*) (Fig. 4, Table 1). Among them, eight tRNAs are encoded by N-strand and the remaining 14 by J-strand. The total length of tRNAs is 1,453 bp, which is shortest among yponomeutoid mitogenomes, which otherwise range from 1,465 bp in *P. xylostella* (KM023645) to 1,488 in *L. malifoliella* (Table 2). As shown in Fig. 4, all tRNAs exhibit typical clover-leaf secondary structure but *trnS1* (AGN) lacks the DHU arm, a feature generally present in all Lepidoptera insects as well as in other metazoan mitogenomes (Garey and Wolstenholme 1989; Lavrov et al. 2000). In tRNAs of the *Y. montanatus* mitogenome, we recognized 22 unmatched base pairs, of which 18 are non-canonical G-U pairs, and the remaining four are mismatched base pairs U-U. The overrepresented non-canonical G-U pairs in tRNAs is commonly present in insect mitogenomes (Salvato et al. 2008; Chen et al. 2016; Chen and Du 2017).

Comparative tRNA analyses among yponomeutoid mitogenomes found that each tRNA structure is highly conserved, including the loss of the DHU arm in *trnS1* (AGN). However, substantial nucleotide variation exists, most of which occurred in the DHU arm and TψC loops (Fig. 4). Interestingly, in *L. malifoliella*, the anticodons for both *trnK* and *trnS1* (AGN) were rarely mutated relative to other yponomeutoid species. For *trnS1* (AGN), the TCT is used instead of routinely used codon GCT. This phenomenon has been recognized in previous reports such as two species of Hepialoidea and three species of Noctuoidea (Li et al. 2018). In *trnK*, the mostly used anticodon CTT was changed to TTT (Wu et al. 2012), which is, to our knowledge, extremely rare in Lepidoptera and insects in general.

Similar to other yponomeutoid mitogenomes, two rRNA genes, *rrnS* and *rrnL*, were recognized in the *Y. montanatus* mitogenome (Fig. 1, Table 1). The *rrnS* is 769 bp long, which is located between *trnV* and A + T-rich region; the *rrnL* is 1,374 bp long, being present between *trnV* and *trnL1*. The lengths of *rrnS* and *rrnL* are comparable to those of other reported yponomeutoid mitogenomes, which are from 770 bp in *L. malifoliella* to 783 bp in *P. xylostella*, and from 1,351 bp in *L. malifoliella* to 1,415 bp in *P. xylostella* (JF911819), respectively.

### Gene overlapping and intergenic regions

**Figure 5.**
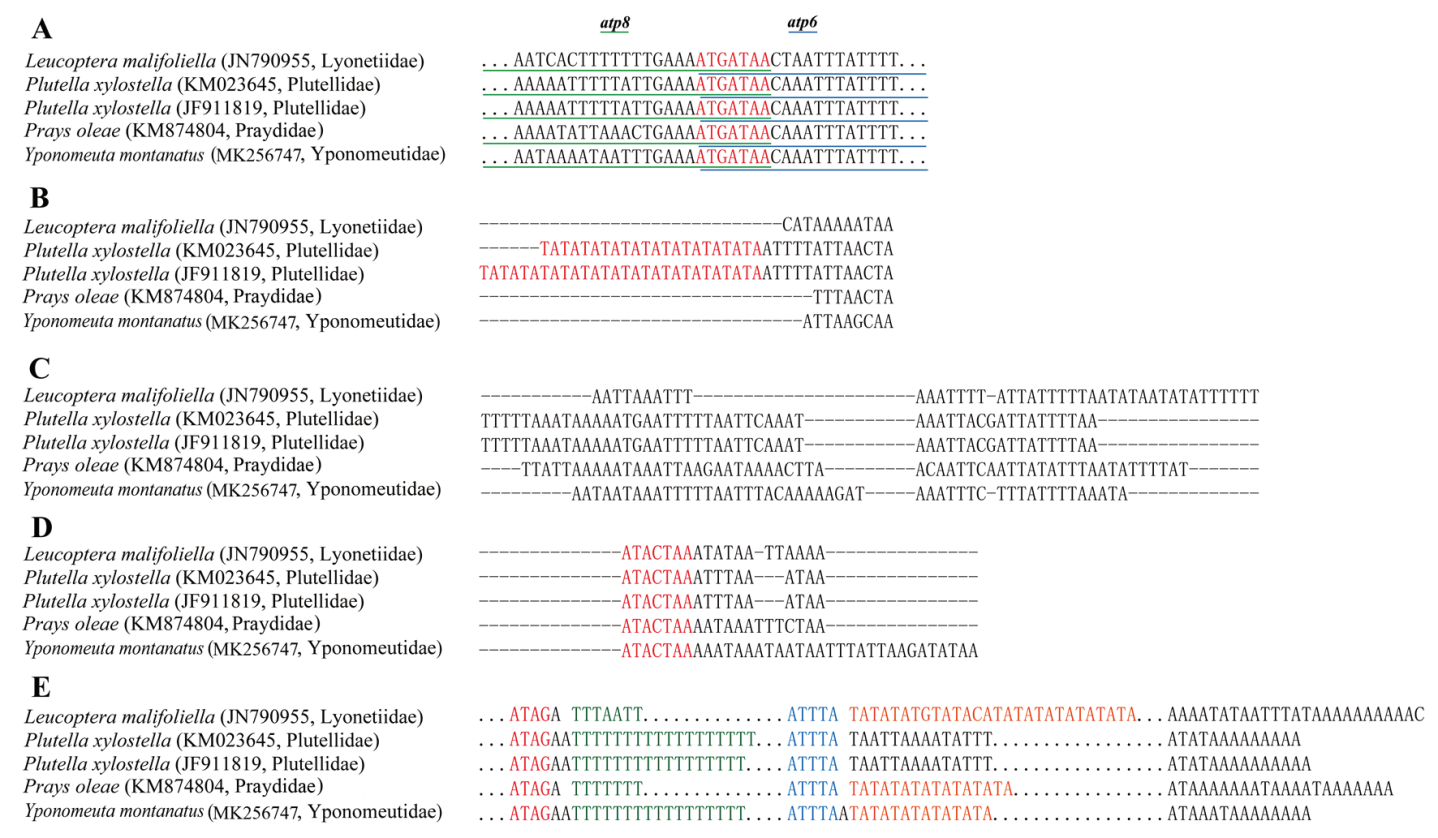
**A** The overlapping region between *atp8* and *atp6*. The nucleotides colored red indicate the sequence of overlapping region; the nucleotides with green underline indicate partial sequence of the *atp8* gene, and the nucleotides with blue underline indicate the partial sequence of the *atp6* gene **B** The intergenic region between *nad6* and *cob.* The microsatellite (TA)_n_ are marked red **C** The intergenic region between *trnQ* and *nad2***D** The intergenic region between *trnS2* and *nad1*. The nucleotides colored red indicate the conserved motif sequence **E** Schematic illustration of the A + T-rich region from all yponomeutoid mitogenomes. The conserved motif ATAG (colored red) and subsequent poly-T stretch (colored green), the conserved motif ATTTA (colored blue) and subsequent (TA)_n_ sequence (colored orange) are emphasized. Dots indicate omitted sequences, and the number of dot is not proportional to nucleotide number of corresponding part.

In the *Y. montanatus* mitogenome, 36 gene overlapping sites were recognized across 13 gene junctions from one to eight bp in length (Table 1). Comparative mitogenome analyses showed that gene overlapping region only between *atp8* and *atp6* is consistently present across reported yponomeutoid mitogenomes. This 7-bp motif of “ATGATAA” (Fig. 5A) is actually a common feature for Lepidoptera and other insects, such as *Taeniopteryx ugola* Ricker & Ross, 1968 (Plecoptera) (Chen and Du 2017).

In addition to the A + T-rich region, a total of 162 intergenic nucleotides across 13 gene junctions from one to 49 bp were identified in *Y. montanatus* mitogenome (Table 1). Among the 13 intergenic regions or site, three are conserved among the reported yponomeutoid mitogenomes, and they are located between the *nad6* and *cob* genes (Fig. 5B), the *trnQ* and *nad2* genes (Fig. 5C), and the *trnS2* and *nad1* genes (Fig. 5D). The one between the *nad6* and *cob* genes ranges from four to 41 bp in length across yponomeutoid mitogenomes. A remarkable feature for this region is that both mitogenomes of *P. xylostella* contain microsatellite (TA)_n_ sequence but with different repeat numbers. *P. xylostella* is an important agricultural pest, and this microsatellite (TA)_n_ sequence may be used as a candidate marker to test the population structure for pest management. The one between the *trnQ* and *nad2* genes exhibits substantial sequence variation (except two sequences for *P. xylostella*) among reported yponomeutoid mitogenomes. This intergenic region is also widely present in other lepidopterans such as those in the Lasiocampidae (Kim et al. 2017) and Noctuidae (Chai and Du 2012), and may even be regarded as an autapomorphy of Lepidoptera (Cao et al. 2014). Also, the intergenic region between *trnS2* and *nad1* is commonly present in insect mitogenomes (Cameron and Whiting 2008). Although the length varies among yponomeutoid mitogenomes, a conserved motif “ATACTAA” could be identified, which has been reported related to mitochondrion transcription (Taanman 1999).

### A + T-rich region

As in other yponomeutoid mitogenomes, the A + T-rich region of the *Y. montanatus* mitogenome is located between the *rrnS* and *trnM* genes (Fig. 1, Table 1). These regions of the published yponomeutoid mitogenomes are remarkably variable in length. The shortest one, consisting of 446 nucleosides, is recognized in the *Y. montanatus* mitogenome. In contrast, the *P. oleae* mitogenome contains the longest one with up to 1,438 bp, and in this region, several tandem repeat elements can be recognized (van Asch et al. 2016). The A + T content of the A + T-rich region among yponomeutoid mitogenomes ranges from 93.1% in *P. xylostella* to 96.3% in *P. oleae*, and all species show the highest A + T content within the whole mitogenome.

Insect mitochondrial A + T-rich region is usually structured in base composition, mainly exhibiting the existence of conserved sequence blocks responsible for mitogenome replication and transcription (Zhang and Hewitt 1997). In the mitogenomes of *Y. montanatus* and other reported yponomeutoids, several conserved sequence blocks could be recognized (Fig. 5E). These blocks include (from 5’ to 3’ end) the motif “ATAG” and subsequent poly-T structure, the motif “ATTTA” and followed microsatellite (TA)_n_ element and an “A”-rich 3’ end upstream of the *trnM* gene. However, in *L. malifoliella* and *P. xylostella*, we did not recognize the poly-T structure and microsatellite (TA)_n_ element, respectively. Also, insect A + T-rich region is generally characterized by the presence of multiple tandem repeat elements (Vila and Björklund 2004). In yponomeutoid mitogenomes, this character can be recognized in *P. oleae* (van Asch et al. 2016) and *L. malifoliella* (Wu et al. 2012). However, in mitogenomes of *Y. montanatus* sequenced herein and *P. xylostella* (Dai et al. 2016), no tandem repeat elements were identified.

### Phylogenetic analyses

To investigate phylogenetic implications of the *Y. montanatus* mitogenome in Yponomeutoidea and Lepidoptera, we constructed the superfamily-level relationships within Lepidoptera using three inference methods and three different datasets.

**Figure 6.**
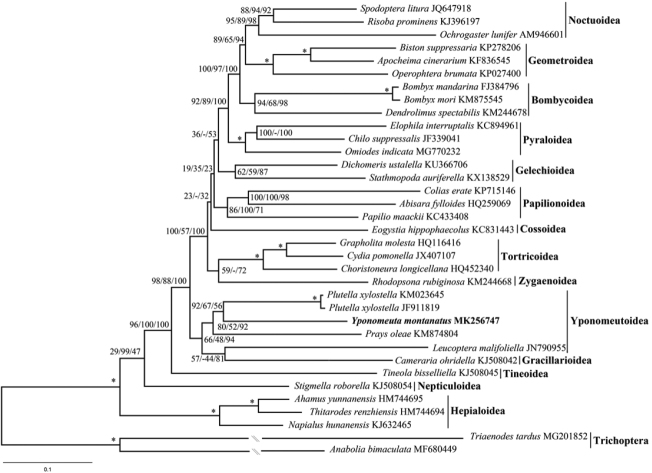
ML tree inferred from RAxML method based on PCG123R dataset. Numbers separated by slash (/) on node represent bootstrap replicates based on PCG123, PCGAA and PCG123R datasets, respectively. The dash (-) represents unrecovered node in ML tree based on the PCG123 or PCGAA dataset.

**Figure 7.**
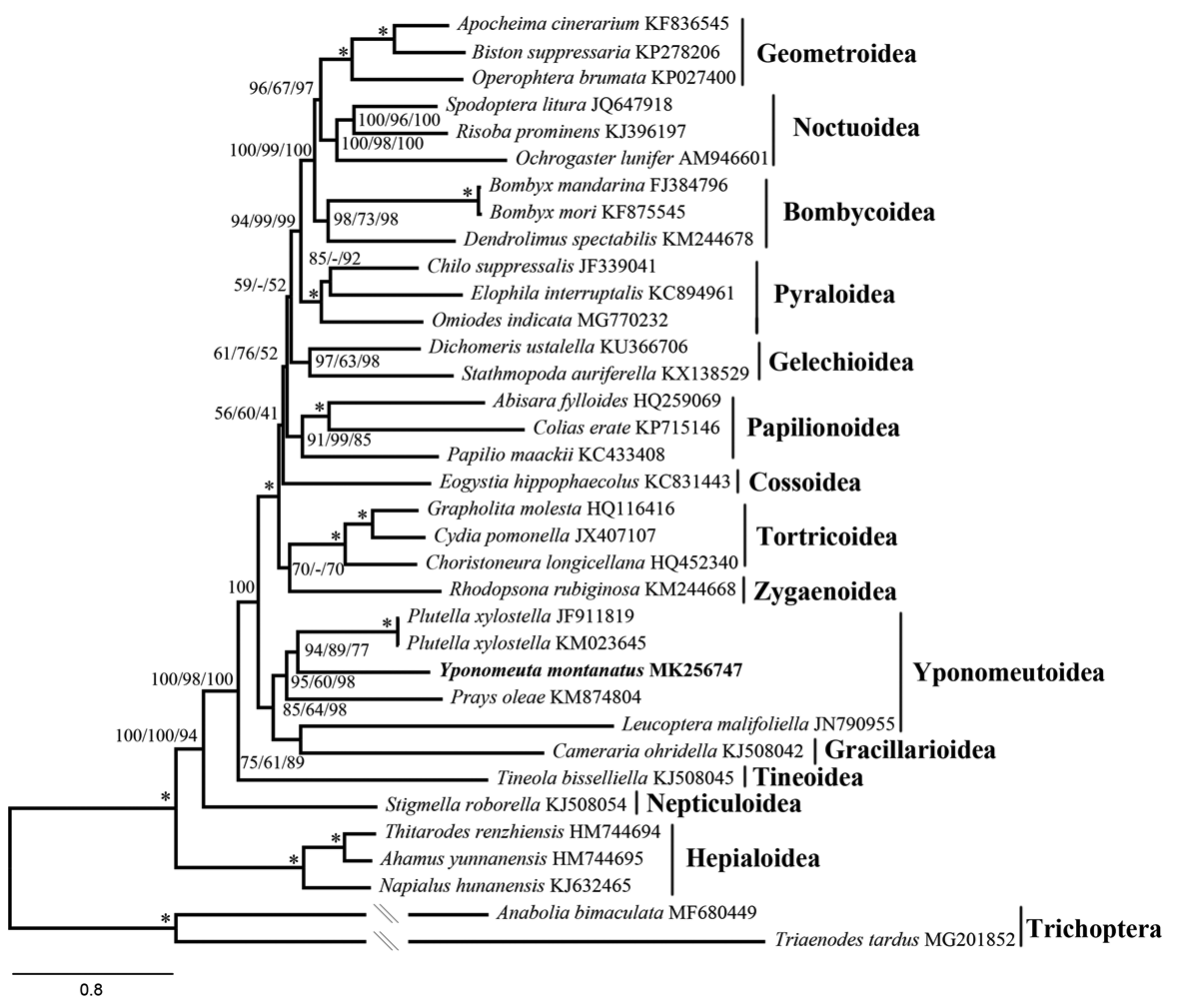
ML tree inferred from IQ-TREE method based on PCG123R dataset. Numbers separated by slash (/) on node represent bootstrap replicates based on PCG123, PCGAA and PCG123R datasets, respectively. The dash (-) represents unrecovered node in ML tree based on the PCG123 or PCGAA dataset.

**Figure 8.**
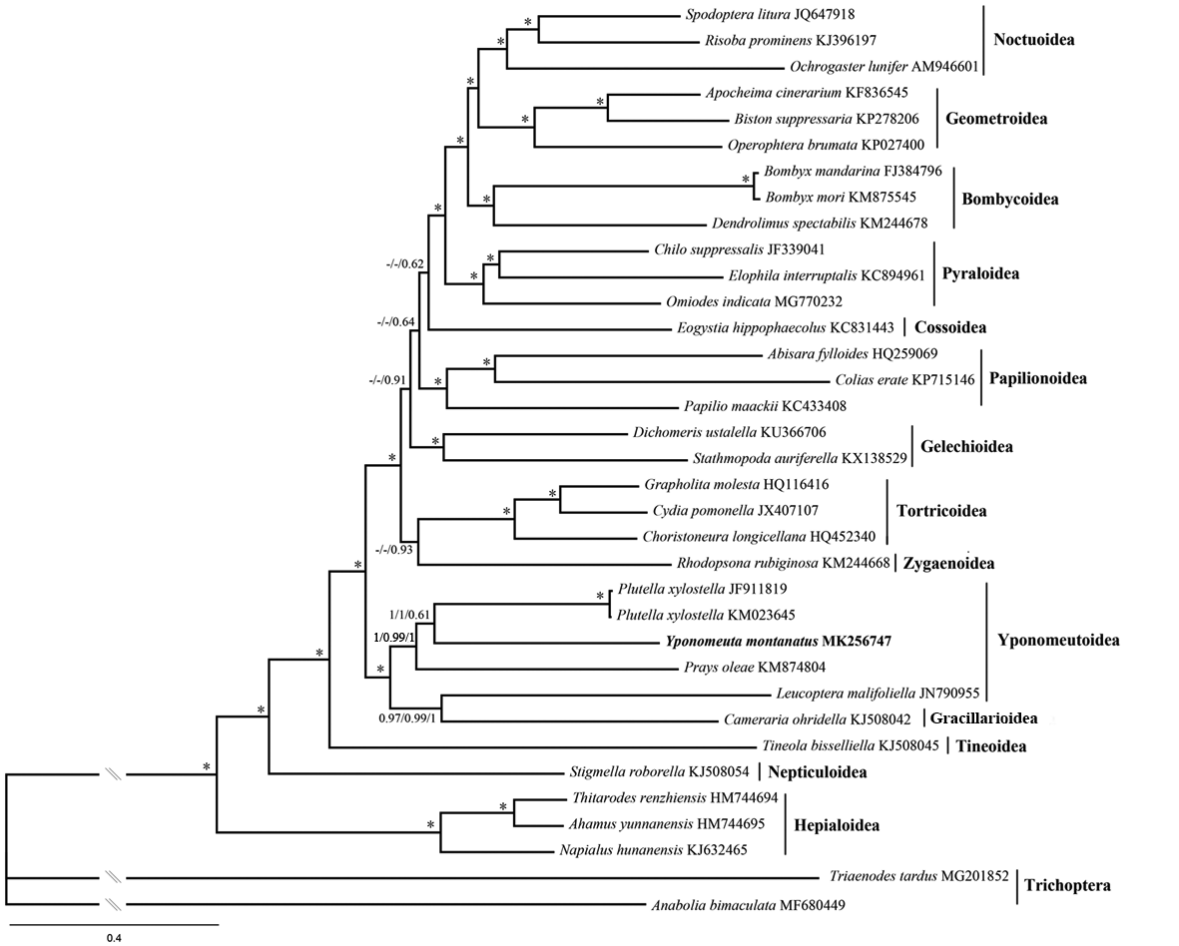
BI tree inferred from MrBayes method based on PCG123R dataset. Numbers separated by slash (/) on node represent posterior probabilities based on PCG123, PCGAA and PCG123R datasets, respectively. The dash (-) represents unrecovered node in BI tree based on the PCG123 or PCGAA dataset.

As shown in Figures 6–8. and S1–S3, relationships among the four yponomeutoid families involved herein were consistently recovered as Lyonetiidae + (Praydidae + (Yponomeutidae + Plutellidae)), which is consistent with that of Sohn et al. (2013) based on multiple-gene data. *Y. montanatus* is nested within Yponomeutoidea, confirming its phylogenetic position using mitogenomic data. In previous studies, the Yponomeutoidea is recovered either sister to Gracillarioidea (Heikkilä et al. 2015) or paraphyletic with respect to Gracillarioidea (Regier et al. 2013; Sohn et al. 2013). Most mitogenome-based phylogenetic studies of Lepidoptera scarcely sampled representatives of Gracillarioidea. As an exception, Timmermans et al. (2014) revealed that Gracillarioidea are nested in the Yponomeutoidea. The same results are recovered in this study. The only representative of Gracillarioidea is consistently sister to *L. malifoliella* in Yponomeutoidea, rendering the Yponomeutoidea paraphyletic.

Regarding the phylogenetic pattern of other superfamilies, mostly identical results were obtained by different analyses, which are also similar to other mitogenome-based studies (Clary and Wolstenholme 1985; Timmermans et al. 2014; Bao et al. 2018). We noticed that the minor topology difference across our analyses mainly occurred in the position of the Papilionoidea, Gelechioidea and Cossoidea. These results are similar to other mitogenome-based studies (Clary and Wolstenholme 1985; Bao et al. 2018) as well as multiple-gene-based study (Heikkilä et al. 2015) where the positions of these superfamilies are unstable or weakly supported, respectively. Within Macroheterocera, Noctuoidea + (Geometroidea + Bombycoidea) were recovered by studies based on various data, such as mitogenome sequences (Kim et al. 2011; Yang et al. 2015), multi-gene sequences (Regier et al. 2013) and 741 genes from transcriptome sequences (Bazinet et al. 2013). Interestingly, our analyses consistently recovered Bombycoidea + (Geometroidea + Noctuoidea), which was identical to that of Kawahara and Breinholt (Kawahara and Breinholt 2014). This result suggests the necessity of extensive phylogenetic investigation when more mitogenomes become available for this clade.

## Supplementary materials

Supplementary material 1 Supplementary Tables S1–S8 Data type: molecular data Explanation note: **Table S1.** List of Lepidoptera species used in phylogenetic analyses. **Table S2.** The best scheme and substitution models for the PCG123R dataset. **Table S3.** The best scheme and substitution models for for the PCG123 dataset. **Table S4.** AT-skew and GC-skew of the *Yponomeuta montanatus* mitogenome. **Table S5.** A + T content (%) in three codon positions in mitochondrial protein-coding genes of reported yponomeutoid mitogenomes. **Table S6.** Codon usage in mitochondrial protein-coding genes of the *Yponomeuta montanatus*. **Table S7.** Start and stop codons of mitochondrial protein-coding genes of four yponomeutoid species. **Table S8.** Evolutionary rates of mitochondrial protein-coding genes among reported yponomeutoid mitogenomes. https://binary.pensoft.net/file/345510This dataset is made available under the Open Database License (http://opendatacommons.org/licenses/odbl/1.0/). The Open Database License (ODbL) is a license agreement intended to allow users to freely share, modify, and use this Dataset while maintaining this same freedom for others, provided that the original source and author(s) are credited.Mingsheng Yang, Bingyi Hu, Lin Zhou, Xiaomeng Liu, Yuxia Shi, Lu Song, Yunshan Wei, Jinfeng Cao

Supplementary material 2 Supplementary Figures S1–S3 Data type: molecular data Explanation note: **Figure S1.**ML trees inferred from RAxML method based on PCG123 (A) and PCGAA (B) datasets. Number on node represents bootstrap replicate. **Figure S2.**ML trees inferred from IQ-TREE method based on PCG123 (A) and PCGAA (B) datasets. Number on node represents bootstrap replicate. **Figure S3.** BI trees inferred from MrBayes method based on PCG123 (A) and PCGAA (B) datasets. Number on node represents posterior probability. https://binary.pensoft.net/file/345511This dataset is made available under the Open Database License (http://opendatacommons.org/licenses/odbl/1.0/). The Open Database License (ODbL) is a license agreement intended to allow users to freely share, modify, and use this Dataset while maintaining this same freedom for others, provided that the original source and author(s) are credited.Mingsheng Yang, Bingyi Hu, Lin Zhou, Xiaomeng Liu, Yuxia Shi, Lu Song, Yunshan Wei, Jinfeng Cao
