# Supplementary material for: First mitochondrial genome from Yponomeutidae (Lepidoptera, Yponomeutoidea) and the phylogenetic analysis for Lepidoptera
Source: Zookeys. 2019 Oct 9;879:137–56. doi: 10.3897/zookeys.879.35101 (PMC6795624; doi:10.3897/zookeys.879.35101)
Supplement: Supplementary material 1 [file zookeys-879-137-s001.docx]

**Table S1.** List of Lepidoptera species used in phylogenetic analyses.

| **Superfamily\order** | **Family** | **Species** | **GenBank accession no.** | **Size (bp)** |
| --- | --- | --- | --- | --- |
| Tortricoidea | Tortricidae | *Grapholita molesta* | HQ116416 | 15,776 |
|  |  | *Cydia pomonella* | JX407107 | 15,253 |
|  |  | *Choristoneura longicellana* | HQ452340 | 15,759 |
| Bombycoidea | Bombycidae | *Bombyx mandarina* | FJ384796 | 15,717 |
|  |  | *Bombyx mori* | KM875545 | 15,666 |
| Lasiocampoidea | Lasiocampidae | *Dendrolimus spectabilis* | KM244678 | 15,411 |
| Geometroidea | Geometridae | *Apocheima cinerarium* | KF836545 | 15,722 |
|  |  | *Operophtera brumata* | KP027400 | 15,748 |
|  |  | *Biston suppressaria* | KP278206 | 15,628 |
| Noctuoidea | Noctuidae | *Spodoptera litura* | JQ647918 | 15,388 |
|  | Nolidae | *Risoba prominens* | KJ396197 | 15,343 |
|  | Notodontidae | *Ochrogaster lunifer* | AM946601 | 15,593 |
| Papilionoidea | Papilionidae | *Papilio maackii* | KC433408 | 15,357 |
|  | Pieridae | *Colias erate* | KP715146 | 15,184 |
|  | Riodinidae | *Abisara fylloides* | HQ259069 | 15,301 |
| Yponomeutoidea | Lyonetiidae | *Leucoptera malifoliella* | JN790955 | 15,646 |
|  | Plutellidae | *Plutella xylostella* | KM023645 | 16,014 |
|  |  | *Plutella xylostella* | JF911819 | 15,646 |
|  | Praydidae | *Prays oleae* | KM874804 | 16,499 |
|  | Yponomeutidae | *Yponomeuta montanatus* | MK256747 | 15.349 |
| Zygaenoidea | Zygaenidae | *Rhodopsona rubiginosa* | KM244668 | 15,248 |
| Pyraloidea | Crambidae | *Omiodes indicata* | MG770232 | 15,367 |
|  |  | *Elophila interruptalis* | KC894961 | 15,351 |
|  |  | *Chilo suppressalis* | JF339041 | 15,395 |
| Gelechioidea | Gelechiidae | *Dichomeris ustalella* | KU366706 | 15,410 |
|  | Oecophoridae | *Stathmopoda auriferella* | KX138529 | 15,456 |
| Cossoidea | Cossidae | *Eogystia hippophaecolus* | KC831443 | 15,431 |
| Gracillarioidea | Gracillariidae | *Cameraria ohridella* | KJ508042 | 15,513 |
| Tineoidea | Tineidae | *Tineola bisselliella* | KJ508045 | 15,661 |
| Nepticuloidea | Nepticulidae | *Stigmella roborella* | KJ508054 | 15,565 |
| Hepialoidea | Hepialidae | *Ahamus yunnanensis* | HM744695 | 15,816 |
|  |  | *Napialus hunanensis* | KJ632465 | 15,301 |
|  |  | *Thitarodes renzhiensis* | HM744694 | 16,173 |
| Trichoptera |  | *Triaenodes tardus* | MG201852 | 14,963 |
|  |  | *Anabolia bimaculata* | MF680449 | 15,048 |

**Table S2.** The best scheme and substitution models for the PCG123R dataset.

| **Partitions** | **Models** | **Genes** |
| --- | --- | --- |
| P1 | GTR + I + G | a6p1, c3p1, cbp1 |
| P2 | TVM + I + G | a6p2, c1p2, c2p2, c3p2, cbp2 |
| P3 | TrN + I + G | n6p3, a6p3, c1p3, c2p3, c3p3, cbp3, n3p3 |
| P4 | GTR + I + G | n6p1, a8p1, a8p2, n2p1, n3p1 |
| P5 | K81uf + G | a8p3, n2p3 |
| P6 | GTR + I + G | c1p1, c2p1 |
| P7 | GTR + I + G | n1p1, n4p1, n4lp1, n5p1 |
| P8 | GTR + I + G | n1p2, n4p2, n4lp2, n5p2 |
| P9 | TIM + I + G | n1p3, n4p3, n4lp3, n5p3 |
| P10 | GTR + G | n2p2, n3p2, n6p2 |
| P11 | GTR + I + G | *rrnS*, *rrnL* |
| P12 | GTR + I + G | tRNAs |

Note: c1–c3, n1–n6, a6, a8 and cb indicate the 13 PCGs; the p1, p2 and p3 indicate the first, second and third codon positions of each PCG respectively.

**Table S3.** The best scheme and substitution models for for the PCG123 dataset.

| **Partitions** | **Models** | **Genes** |
| --- | --- | --- |
| P1 | GTR + I + G | a6p1, c2p1,c3p1, cbp1 |
| P2 | TVM + I + G | a6p2, c1p2, c2p2, c3p2, cbp2 |
| P3 | TrN + I + G | n6p3, a6p3, c1p3, c2p3, c3p3, cbp3, n3p3 |
| P4 | GTR + I + G | n6p1, a8p1, a8p2, n2p1, n3p1 |
| P5 | K81uf + G | a8p3, n2p3 |
| P6 | GTR + I + G | c1p1, c2p1 |
| P7 | GTR + I + G | n1p1, n4p1, n4lp1, n5p1 |
| P8 | GTR + I + G | n1p2, n4p2, n4lp2, n5p2 |
| P9 | GTR + I + G | n1p3, n4p3, n4lp3, n5p3 |
| P10 | GTR + G | n2p2, n3p2, n6p2 |

Note: c1–c3, n1–n6, a6, a8 and cb indicate the 13 PCGs; the p1, p2 and p3 indicate the first, second and third codon positions of each PCG respectively.

**Table S4.** AT-skew and GC-skew of the *Yponomeuta montanatus* mitogenome.

| **Feature** | **Size (bp)** | **AT-skew** | **GC-skew** |
| --- | --- | --- | --- |
| Mitogenome | 15,349 | 0.0037 | –0.164 |
| PCGs | 11,145 | –0.142 | 0.039 |
| PCGs-1st | 3,715 | –0.023 | 0.247 |
| PCGs-2nd | 3,715 | –0.365 | –0.098 |
| PCGs-3rd | 3,715 | –0.071 | –0.121 |
| PCGs-J | 6,879 | –0.114 | –0.088 |
| PCGs-J-1st | 2,293 | 0.022 | 0.15 |
| PCGs-J-2nd | 2,293 | –0.338 | –0.19 |
| PCGs-J-3rd | 2,293 | –0.052 | –0.559 |
| PCGs-N | 4,266 | –0.188 | 0.277 |
| PCGs-N-1st | 1,422 | –0.088 | 0.427 |
| PCGs-N-2nd | 1,422 | –0.41 | 0.073 |
| PCGs-N-3rd | 1,422 | –0.1 | 0.639 |
| tRNAs | 1,453 | 0.027 | 0.188 |
| tRNAs-J | 928 | 0.035 | 0.076 |
| tRNAs-N | 525 | 0.013 | 0.384 |
| rRNAs | 2,143 | 0.0094 | 0.333 |
| *rrnS* | 769 | 0.029 | 0.315 |
| *rrnL* | 1,374 | –0.0012 | 0.342 |
| A + T-rich region | 446 | –0.025 | –0.316 |

**Table S5.** A + T content (%) in three codon positions in mitochondrial protein-coding genes of reported yponomeutoid mitogenomes.

| **Species** | **A + T content (%) in codon positions** | | |
| --- | --- | --- | --- |
|  | **1** | **2** | **3** |
| *Leucoptera malifoliella* | 76.4 | 71.7 | 93.9 |
| *Plutella xylostella* (KM023645) | 74.6 | 70.5 | 93.2 |
| *Plutella xylostella* (JF911819) | 74.5 | 70.3 | 93.2 |
| *Prays oleae* | 74.5 | 70.5 | 92.2 |
| *Yponomeuta montanatus* | 74.6 | 70.4 | 93.5 |

**Table S6.** Codon usage in mitochondrial protein-coding genes of the *Yponomeuta montanatus* mitogenome.

| **Codon** | **Count** | **RSCU** | **Codon** | **Count** | **RSCU** | **Codon** | **Count** | **RSCU** | **Codon** | **Count** | **RSCU** |
| --- | --- | --- | --- | --- | --- | --- | --- | --- | --- | --- | --- |
| UUU (F) | 364 | 1.9 | UCU (S) | 105 | 2.68 | UAU (Y) | 170 | 1.78 | UGU (C) | 34 | 1.94 |
| UUC (F) | 19 | 0.1 | UCC (S) | 10 | 0.25 | UAC (Y) | 21 | 0.22 | UGC (C) | 1 | 0.06 |
| UUA (L) | 495 | 5.37 | UCA (S) | 86 | 2.19 | UAA (*) | 11 | 2 | UGA (W) | 93 | 1.88 |
| UUG (L) | 9 | 0.1 | UCG (S) | 3 | 0.08 | UAG (*) | 0 | 0 | UGG (W) | 6 | 0.12 |
| CUU (L) | 33 | 0.36 | CCU (P) | 83 | 2.63 | CAU (H) | 62 | 1.85 | CGU (R) | 18 | 1.38 |
| CUC (L) | 4 | 0.04 | CCC (P) | 14 | 0.44 | CAC (H) | 5 | 0.15 | CGC (R) | 1 | 0.08 |
| CUA (L) | 12 | 0.13 | CCA (P) | 29 | 0.92 | CAA (Q) | 61 | 1.94 | CGA (R) | 32 | 2.46 |
| CUG (L) | 0 | 0 | CCG (P) | 0 | 0 | CAG (Q) | 2 | 0.06 | CGG (R) | 1 | 0.08 |
| AUU (I) | 411 | 1.95 | ACU (T) | 69 | 1.83 | AAU (N) | 239 | 1.82 | AGU (S) | 25 | 0.64 |
| AUC (I) | 11 | 0.05 | ACC (T) | 8 | 0.21 | AAC (N) | 23 | 0.18 | AGC (S) | 4 | 0.1 |
| AUA (M) | 278 | 1.85 | ACA (T) | 73 | 1.93 | AAA (K) | 102 | 1.87 | AGA (S) | 81 | 2.06 |
| AUG (M) | 22 | 0.15 | ACG (T) | 1 | 0.03 | AAG (K) | 7 | 0.13 | AGG (S) | 0 | 0 |
| GUU (V) | 62 | 1.94 | GCU (A) | 84 | 2.71 | GAU (D) | 51 | 1.76 | GGU (G) | 50 | 0.99 |
| GUC (V) | 0 | 0 | GCC (A) | 6 | 0.19 | GAC (D) | 7 | 0.24 | GGC (G) | 2 | 0.04 |
| GUA (V) | 62 | 1.94 | GCA (A) | 31 | 1 | GAA (E) | 66 | 1.71 | GGA (G) | 113 | 2.24 |
| GUG (V) | 4 | 0.13 | GCG (A) | 3 | 0.1 | GAG (E) | 11 | 0.29 | GGG (G) | 37 | 0.73 |

**Table S7.** Start and stop codons of mitochondrial protein-coding genes of four yponomeutoid species.

| **Species** | **Start codon\stop codon** | | | | | | | | | | | | |
| --- | --- | --- | --- | --- | --- | --- | --- | --- | --- | --- | --- | --- | --- |
|  | ***atp6*** | ***atp8*** | ***cox1*** | ***cox2*** | ***cox3*** | ***cob*** | ***nad1*** | ***nad2*** | ***nad3*** | ***nad4*** | ***nad4l*** | ***nad5*** | ***nad6*** |
| *L. malifoliella* | ATG\TAA | ATT\TAA | CGA\TAA | ATA\T | ATG\TAA | ATG\TAA | ATA\TAA | ATT\T | ATT\TAA | ATG\TAA | ATG\TAG | ATT\T | ATA\TAG |
| *P. xylostella*  (KM023645) | ATG\TAA | ATC\TAA | CGA\TAA | ATG\T | ATG\TAA | ATG\TAA | ATG\TAA | ATT\TAA | ATG\TAA | ATG\T | ATG\TAA | ATT\TAA | ATT\TAA |
| *P. xylostella*  (JF911819) | ATG\TAA | ATC\TAA | CGA\TAA | ATG\T | ATG\TAA | ATG\TAA | ATG\TAA | ATT\T | ATG\TAA | ATG\T | ATG\TAA | ATT\TAA | ATT\TAA |
| *P. oleae* | ATG\TAA | ATT\TAA | CGA\TAA | ATG\T | ATG\TAA | ATG\TAA | ATG\TAA | ATC\TAA | ATG\TAA | ATG\TAA | ATG\TAA | ATT\TAA | ATT\T |
| *Y. montanatus* | ATG\TAA | ATT\TAA | CGA\TAA | ATG\T | ATG\TAA | ATG\TAA | ATG\TAA | ATT\TAA | ATT\TAA | ATG\T | ATG\TAA | ATT\TAA | ATT\TAA |

**Table S8.** Evolutionary rates of mitochondrial protein-coding genes among reported yponomeutoid mitogenomes.

| **PCGs** | **Alignment size (bp)** | **Nucleotide diversity** | **Ka/Ks** |
| --- | --- | --- | --- |
| *atp6* | 675 | 0.1671 | 0.2286 |
| *atp8* | 171 | 0.2184 | 0.5662 |
| *cox1* | 1533 | 0.1205 | 0.1103 |
| *cox2* | 684 | 0.1233 | 0.1474 |
| *cox3* | 789 | 0.1611 | 0.1655 |
| *cob* | 1152 | 0.146 | 0.1585 |
| *nad1* | 939 | 0.1747 | 0.3841 |
| *nad2* | 1017 | 0.2033 | 0.3543 |
| *nad3* | 351 | 0.1792 | 0.3082 |
| *nad4* | 1338 | 0.1787 | 0.4171 |
| *nad4l* | 291 | 0.1454 | 0.5079 |
| *nad5* | 1725 | 0.1594 | 0.5079 |
| *nad6* | 531 | 0.2593 | 0.4994 |

Note: Ka/Ks, the ratio of nonsynonymous substitution (Ka) to synonymous substitution (Ks).
